# Evaluation and In Vitro Study of an Electrospun Bone Tissue Membrane for Bone Regeneration: A Novel Perspective

**DOI:** 10.7759/cureus.52830

**Published:** 2024-01-23

**Authors:** Nazurudeen Jabeen, Anitha Roy, Rethinam Senthil

**Affiliations:** 1 Department of Pharmacology, Saveetha Dental College and Hospitals, Saveetha Institute of Medical and Technical Sciences, Chennai, IND

**Keywords:** synthetic polymer, tissue engineering, biocompatibility, gelatin, electrospun membrane

## Abstract

Objectives

In the present study, electrospun bone tissue membrane (EBTM) was prepared using polyvinylidene fluoride (PVDF), gelatin (gel), and demineralized bone matrix (DBM) by electrospinning method for its potential application in bone tissue regeneration.

Materials and methods

The prepared EBTM was evaluated using high-resolution scanning electron microscopy (HR-SEM), energy-dispersive X-ray spectroscopy (EDX; Silicon Drift 2017, USA), thermogravimetric analysis (TGA), and mechanical properties such as tensile strength (MPa), elongation at break (%), flexibility (%), and water absorption (%). In vitro bioactivity testing of EBTM using simulated body fluid (SBF) was performed after 14 days of immersion. Cell viability was tested using human osteoblast-like cells (MG-63) to prove biocompatibility.

Results

EBTM had superior surface morphology, thermal stability, and mechanical strength. The mechanical properties of EBTM were promising, enabling its use in tissue engineering. Bioactivity test showed that the EBTM surface developed calcium (Ca) and phosphate (P) after 14 days of being immersed in SBF. Additionally, a biocompatibility investigation revealed that EBTM was covered with more viable cells.

Conclusion

EBTM with sufficient mechanical strength, thermal stability, surface morphology, Ca deposition, and biocompatibility could serve as a plausible material for bone tissue engineering (skin, ligament, cartilage, and bone).

## Introduction

In recent years, a lot of effort has been put into developing biodegradable and biocompatible scaffolds that resemble the structural and functional properties of extracellular matrix (ECM) [1,2] for bone tissue engineering and regenerative medicine. The choice of appropriate material to build scaffolds that act as a bed for bone formation is one of the challenges in bone tissue engineering. Gelatin (Gel) is a biodegradable biopolymer that is derived from fish bone using an acidic process. Natural polymers (collagen and Gel) have been widely used for the preparation of electrospun membranes. In the present work, a biodegradable and biocompatible protein such as Gel was selected for scaffold development due to its lack of antigenicity in physiological environments when compared to collagen. Fish bone waste contains Gel, one of the most popular biopolymers derived from animal proteins [3]. Gel is a highly effective polymer for electrospinning used in the manufacture of nanometric-size fibers regardless of environmental temperature and humidity fluctuations; as a result, it was employed as a basis for the development of bioactive electrospun fibers. Several types of electrospun Gel are used in bone tissue engineering [4].

The electrospinning technology has been used as an accessible and efficient approach for producing nanofibres with numerous applications in medicine and pharmaceutics. An electrospun membrane comprising polymer fibers with sizes ranging from 100 nanometers to micrometers was produced using electrospinning techniques. Fibrous scaffolds can stimulate cell adhesion and proliferation due to their structural resemblance to ECM [5,6]. Electrospun scaffold-based study demonstrated that electrospun scaffolds improve the growth rate, cellular activity, and bone healing when compared with micron-sized porous materials. Allografts and autografts stimulate bone production, and metallic implants require their removal as using allografts has a high risk of morbidity and disease transmission [7]. All the limitations related to the use of allografts, autografts, and metal implants can be overcome by tissue engineering.

Demineralized bone matrix (DBM) is a naturally occurring polymer mixture from which the mineral has been removed; it is taken from bone specimens. The protein components of bone such as adhesion ligands and osteoinductive signals are present in DBM. The anabolic and catabolic processes required to maintain strong bones are performed by osteoblasts in this organic protein network. Over the last three decades, there has been a substantial rise in the utilization of DBM in orthopedic surgery, leading to its remodeling and promoting mineralization [8]. The development of electrospun bone tissue membranes (EBTMs) for bone tissue engineering has involved an extensive examination of natural and synthetic polymers.

The objective of this study was to develop an EBTM using polyvinylidene fluoride (PVDF; synthetic polymer), Gel (natural polymer), and DBM. The results of the calcium (Ca)/phosphate (P) surface mineralization study of the developed bone tissue membrane and mechanical, biocompatibility, and physicochemical analysis would support improved bone tissue regeneration (dental connective tissue).

## Materials and methods

Preparation of Gel

Gel was prepared using Bluefin Trevally (*Caranx melampygus*) fish bone according to the method in [9]. Briefly, to remove noncollagenous proteins, the bone was cut into small pieces and soaked in 0.1 M sodium hydroxide (NaOH) solution (1:30 w/v); the NaOH solution was changed twice every 2 h. The NaOH-treated bone was decalcified using 0.4 ethylenediaminetetraacetic acid disodium salt (EDTA-2Na) solution (0.5:15 w/v). For the extraction of the Gel, the decalcified fish bone was soaked in deionized water for 8 h at 55°C while being constantly agitated. The bone sample was then centrifuged for 10 min at 8000 rpm to remove the particles. The Gel supernatant was freeze-dried using a high-pressure dryer.

Preparation of DBM

The fish bone waste was collected from a nearby fish market (Chennai, India). It was cleaned and boiled in water at 100°C for 10 min. The bones were crushed into a paste with the help of a grinder. The paste was made into a dry powder by using the microwave drier at 90°C for 100 min. The powder was further dried for 24 h at 60°C using a dry-heat oven. Subsequently, this powder was put through a 12 h high-energy ball milling process to generate a fine powder. Powder samples were stored in a desiccator to maintain their dry state. The fine dry powder was subjected to a reflux deproteinization process with the use of 5% potassium hydroxide (KOH), which helped in converting the protein and Ca in the fish bone.

Preparation of EBTM

The electrospinning solution denoted as PVDF:Gel:DBM was fabricated using 8.0 wt% PVDF with de-ionized water using a stirrer at 50°C for 1 h. The PVDF solution was then mixed with 1.0 g of Gel, 2.0 wt% DBM, and 0.5 wt% glacial acetic (CH₃COOH), followed by vigorous stirring for 2 h. The resulting solution was electrospun at a voltage of 15-20 kV with a tip-to-collector distance of 15 cm and a flow rate of 0.5 ml/min. The spin-coated film was collected with a flat sheet of aluminum foil. The electrospinning condition was kept at 30°C, and the film thickness was kept within the range of 0.15-0.05 mm. Two additional membranes were electrospun: PVDF (8 wt%) and PVDF (8 wt%):Gel (1.0 g).

Bioactivity test

We evaluated the apatite formation capacity of EBTM by immersing 2x2 cm square samples in 10X SBF. The samples were completely immersed in 4 ml of 10X SBF and kept in an incubator at 37°C. After day 1 and day 14, EBTM was removed from SBF and allowed to dry. High-resolution scanning electron microscopy (HR-SEM) images were captured to look at the formation of Ca/P crystals on the outermost layer of EBTM fibers. After day 14, the Ca/P ratio was also measured using metal mapping and energy-dispersive X-ray spectroscopy (EDX; Silicon Drift 2017, USA).

Cell viability

The 3-(4,5-dimethylthiazol-2-yl)-2,5-diphenyl tetrazolium bromide (MTT) assay was used to evaluate the cell viability of EBTM. The EBTM was sterilized for 1 h with ultraviolet (UV) light before being washed with phosphate-buffered saline (PBS), which was applied to a 96-well cell culture platform at pH 2. MG-63 human osteoblast cells (5×10^5^ cells/ml: P7) were inoculated into each well and incubated for 24, 48, and 78 h under CO_2_ at 37°C, respectively. Following incubation, the cells were treated for 3 h with MTT (0.5 mg/ml). The crystals generated were dispersed using dimethylsulfoxide (DMSO). The testing was done at 570 nm with Spectrum Maximum M4.

## Results

Characterization of Gel and DBM

**Figure 1:**
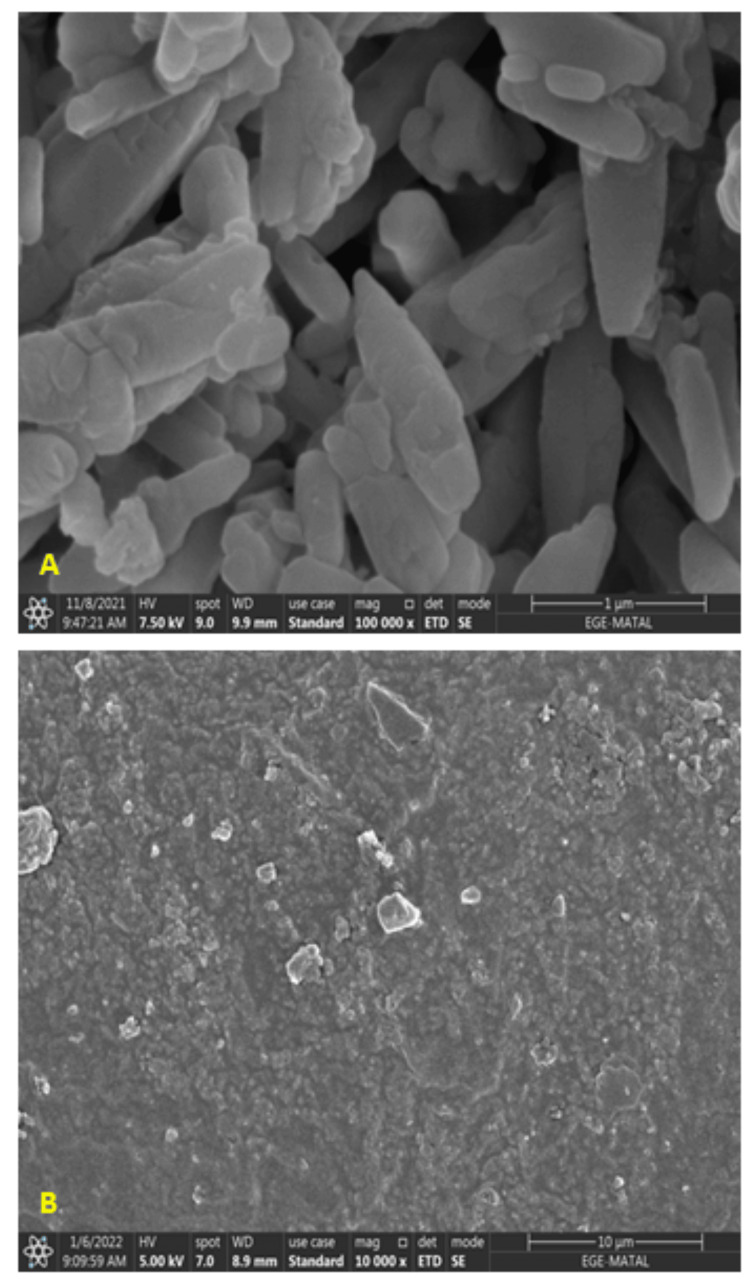
The figure depicts HR-SEM images of (A) Gel and (B) DBM HR-SEM: High-resolution scanning electron microscopy; Gel: Gelatin; DBM: Demineralized bone matrix

The HR-SEM image of the Gel is displayed in Figure 1A. The morphology of the Gel was thin and the surface smooth. Figure 1B shows the DBM microstructure as determined by HR-SEM. This DBM had a cylindrical shape and a porous structure. The DBM pore size was not uniform and had an average size of less than 1 μm. The proportion of Ca/P crystals that nucleated on the DBM was not significant under these circumstances. Since oxygen transfer is one of the advantages of DBM, this porosity might be solved by using an electrospun nano-filler.

Characterization of EBTM

**Figure 2:**
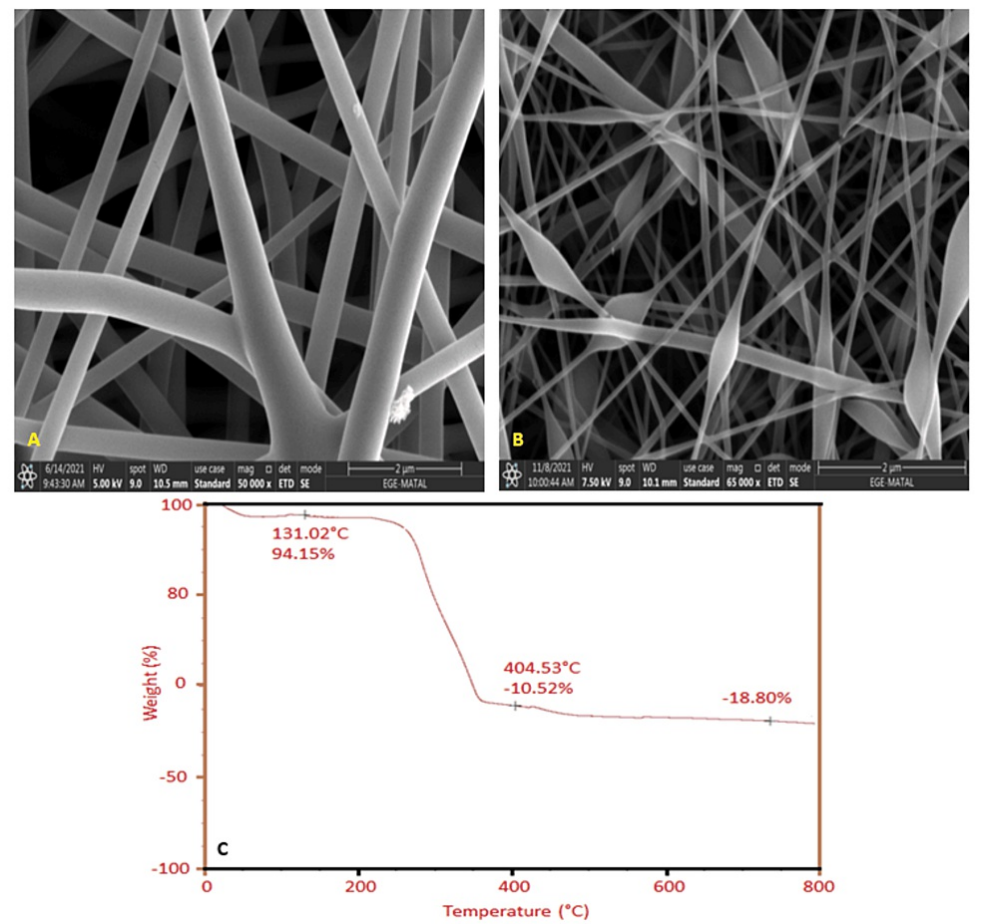
The figure depicts (A) HR-SEM image of PVDF:Gel, (B) HR-SEM image of PVDF:Gel:DBM, and (C) TGA of PVDF:Gel:DBM HR-SEM: High-resolution scanning electron microscopy; PVDF: Polyvinylidene fluoride; Gel: Gelatin; DBM: Demineralized bone matrix; TGA: Thermogravimetric analysis

The HR-SEM images of PVDF:Gel and PVDF:Gel:DBM are displayed in Figure 2A-2B, respectively. Due to the presence of Gel and DBM on the PVDF (EBTM), surface fibers had an average diameter larger than that of the PVDF:Gel membrane. The membrane was in the nano-size range between 100-200 nm. It was discovered that the membrane had a porosity of 75-80%. Figure 2C displays the TGA of EBTM. The TGA curve of EBTM indicates that the evaporation of water molecules at about 100°C causes 12% weight loss. Due to the PVDF, Gel, and DBM breaking down, the EBTM begins to degrade from 200°C to 400°C and lose about 80% of its initial weight.

Mechanical properties

The mechanical properties of PVDF, PVDF:Gel, and PVDF:Gel:DBM are shown in Table 1. Tensile strength, elongation at break, flexibility, water absorption, and desorption characteristics were compared among the prepared PVDF, PVDF:Gel, and PVDF:Gel:DBM (EBTM) membranes to evaluate their mechanical strength. Table 1 demonstrates that EBTM had the highest mechanical strength in comparison to the other two membranes.

**Table 1:** Mechanical properties of PVDF, PVDF:Gel, and PVDF:Gel:DBM PVDF: Polyvinylidene fluoride; Gel: Gelatin; DBM: Demineralized bone matrix

Samples	Tensile Strength (MPa)	Elongation at break (%)	Flexibility (%)	Water absorption (%)
PVDF	23.56+1.06	27.65+0.54*	5.35+0.76*	35.98+0.66
PVDF:Gel	24.73+1.31*	28.67+0.71	6.72+0.53	34.56+0.82*
PVDF:Gel:DBM	25.15+1.56	29.35+0.57*	7.91+0.86	33.67+0.12*

Bioactivity analysis

**Figure 3:**
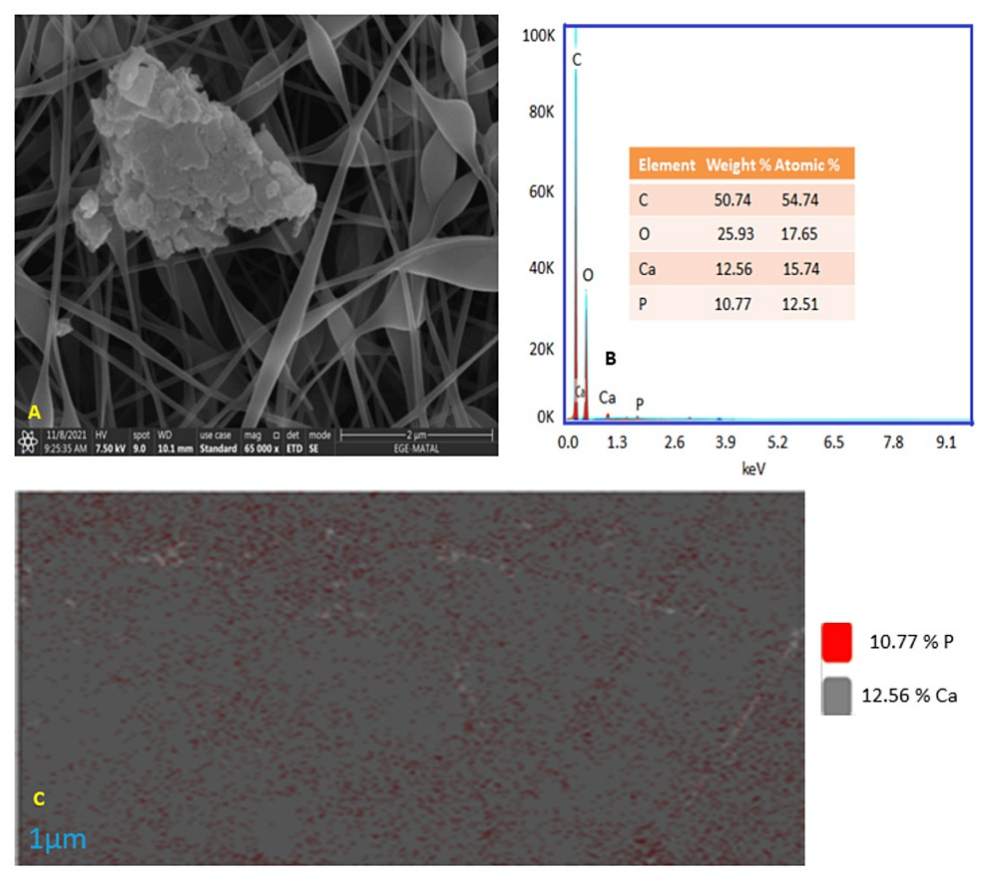
The figure depicts (A) HR-SEM image, (B) EDX analysis, and (C) Elemental mapping of PVDF:Gel:DBM after SBF treatment HR-SEM: High-resolution scanning electron microscopy; EDX: Energy-dispersive X-ray spectroscopy; PVDF: Polyvinylidene fluoride; Gel: Gelatin; DBM: Demineralized bone matrix; SBF: Simulated body fluid

The image obtained by HR-SEM shows the EBTM after it was immersed in SBF (Figure 3A). The HR-SEM image indicates that the EBTM surface has been completely covered by Ca/P crystal formation. This feature could be seen in bone growth as it encourages the production of an amorphous cylindrical agglomeration on the surface membrane. Figure 3B displays the results of EDX, which show that the EBTM, adhering to the SBF treatment, has a Ca/P crystalline peak. Figure 3C shows the elemental analysis, which confirmed the presence of Ca/P.

Biocompatibility study

**Figure 4:**
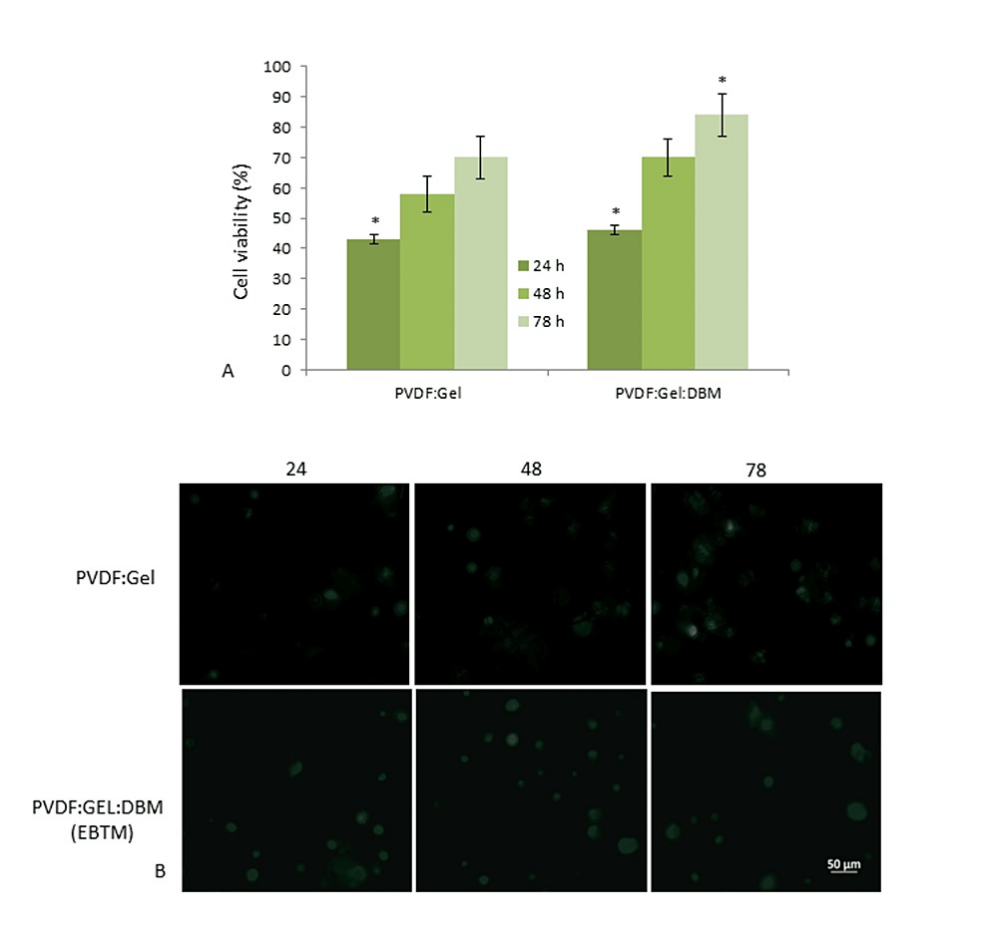
The figure depicts (A) MTT assay of human osteoblast-like (MG-63) cells on PVDF:Gel and PVDF:Gel:DBM; (B) Fluorescence micrographs (20X) of MG-63 cells cultured at 24, 48 and 78 h The asterisks (*) indicate statistically significant differences compared to the control p<0.05. MTT: 3-(4,5-dimethylthiazol-2-yl)-2,5-diphenyl tetrazolium bromide; PVDF: Polyvinylidene fluoride; Gel: Gelatin; DBM: Demineralized bone matrix

The cell viability percentage on PVDF:Gel and PVDF:Gel:DBM membranes is shown in Figure 4A. The results depict the cell viability of MG-63 human osteoblast cells on PVDF:Gel and PVDF:Gel:DBM at 24, 48, and 78 h. The morphological image of MG-63 human osteoblast cells observed on the EBTM was evaluated through fluorescence microscopy, as shown in Figure 4B. The results indicate that the EBTM exhibits non-toxic, biocompatible, and 100% viability to cells. The cells on the EBTM increased significantly faster after 78 h than the cells on the PVDF:Gel, indicating that the membrane encouraged cell growth. According to the findings, the bioactive membrane phase may be crucial in modulating the cellular reaction to the membrane.

## Discussion

Polymeric membranes provide the appropriate environment for bone tissue regeneration. The biostability of a tissue scaffold can be determined by closely studying its three key elements: elasticity, strength, and absorption of biomolecules into the material surface [10]. Mechanical properties should be preserved during implant, especially when it comes to significant load-bearing elements like bones, and the combined membrane should have better mechanical properties, resistance to degradation, and affinity for biological components. Both synthetic and natural polymers are used to make certain important nanofibrous scaffolds for bone tissue engineering applications. Electrospun membranes improve osteoconduction and osteointegration significantly, improving the osteogenic cells to identify nanoscale functions and minerals and enhance cellular interaction and proteins [11]. As a result, in this work, electrospinning was selected to generate fibers with diameters in the nanoscale range. The observed diameter of PVDF nanofibers was found in agreement with that presented in previous literature [12]. The electrospun membrane is a dynamic environment that regulates cellular behavior and tissue functionality. Our previous studies focused on the use of electrospun scaffolds made of synthetic as well as natural polymers as nanomaterial for bone tissue engineering [13,14].

Due to the inclusion of inorganic components from DBM, the EBTM has high thermal stability. This is consistent with research findings [15] from the investigation on DBM. The addition of Gel resulted in a smooth surface shape, as reported in [16]. The reported value for cellular attachment and proliferation suggests that the surface is smooth. The porosity (73.9%) was appropriate for bone tissue engineering applications, according to [17]. "Nanonets" that form a web-like shape are lightweight fibers with a width of 15-30 nm. The Gel and DBM containing PVDF membrane comprised Ca and P components, which were visible during EDX analysis. Due to the extremely low concentration, their peaks were insignificant when compared to the primary polymer elements (carbon and oxygen). In vitro bioactivity studies showed that the EBTM included a surface covered with Ca and P after 14 days of immersion, as evidenced by EDX analysis. This result was consistent with previous work [18], which reported that apatite production occurred when using DBM. The mechanical and physical characteristics of membranes that are biocompatible, strong, and biodegradable, as well as chemically inert and having highly functional surfaces, are excellent for use in tissue regeneration. These membranes serve as a biomimetic model for controlling the formation of cellular interactions and tissue regeneration.

According to the findings of the tensile test, the EBTM membrane had the maximum strength when compared to other scaffolds, surpassing the values published in [19]. Of the examined membranes, the EBTM had a high water absorption value. PVDF in the EBTM exhibits a strengthening function. In contrast, fibers containing Gel exhibit random network shape remodeling in the bone [20]. The DBM present in dental implants had significant amounts of hydroxyl functional groups that enhanced cell viability and cell attachment [21]. The biocompatibility of the bone implant material is one of the most significant characteristics for the safe and effective use of these implants in clinical practice [22].

Based on the findings of this research, it could be stated that electrospinning techniques are important for developing EBTM and for assessing their features in the area of bone tissue regeneration (connective dental tissue and orthopedic field). These membranes could offer superior biological, physical, and mechanical properties compared to synthetic film equivalents. Furthermore, it is suggested that in vivo studies be conducted to assess the therapeutic function of the EBTM.

In short, limitations are associated with the existing procedures relating to the preparation of EBTM. The major drawback of EBTM is the more hydrophilic condition of DBM. Hence, we are continuing to work on bone tissue membranes, and we hope to reduce water absorption.

## Conclusions

The current study successfully developed EBTM through the use of electrospinning techniques. The optimized electrospinning parameters resulted in a smooth and uniform nanofibrous scaffold. The developed EBTM exhibited a significant value of elongation at break and tensile strength. The EBTM has shown promise for application in bone tissue healing because morphological tests have indicated that they could preserve their web-like structure. Through cytotoxicity experiments, the safety and biocompatibility of EBTM with human osteoblast-like cells (MG-63) were determined. Bone apatite formation was achieved by treatment with accelerated bone tissue engineering, as demonstrated by the bioactivity test. The developed EBTM demonstrated potential for more study on animals and clinical trials, which would enable its commercialization.
